# Applications of phase change materials in smart drug delivery for cancer treatment

**DOI:** 10.3389/fbioe.2022.991005

**Published:** 2022-09-12

**Authors:** Jianfeng Bao, Hui Tu, Jing Li, Yijia Li, Shan Yu, Jingpi Gao, Kun Lei, Fengshou Zhang, Jinghua Li

**Affiliations:** ^1^ School of Medical Technology and Engineering, Henan University of Science and Technology, Luoyang, China; ^2^ Office of Science & Technology, Henan University of Science and Technology, Luoyang, China

**Keywords:** PCMs, drug delivery, drug encapsulation, controlled release, thermochemical therapy

## Abstract

Phase change materials (PCMs) are materials that are stimulated by the external enthalpy change (temperature) to realize solid-liquid and liquid-solid phase transformation. Due to temperature sensitivity, friendly modification, and low toxicity, PCMs have been widely used in smart drug delivery. More often than not, the drug was encapsulated in a solid PCMs matrix, a thermally responsive material. After the trigger implementation, PCMs change into a solid-liquid phase, and the loading drug is released accordingly. Therefore, PCMs can achieve precise release control with different temperature adjustments, which is especially important for small molecular drugs with severe side effects. The combination of drug therapy and hyperthermia through PCMs can achieve more accurate and effective treatment of tumor target areas. This study briefly summarizes the latest developments on PCMs as smart gate-keepers for anti-tumor applications in light of PCMs becoming a research hot spot in the nanomedicine sector in recent years.

## 1 Introduction

**FIGURE 1:**
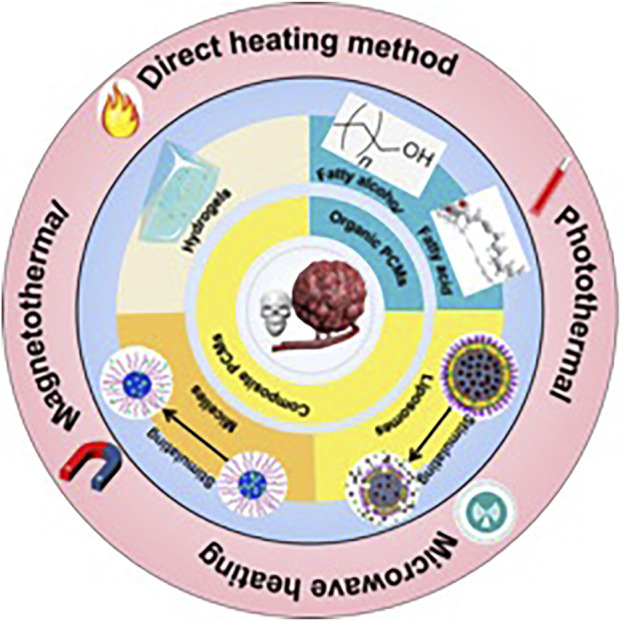
Types of PCMs used for intelligent antitumor administration in nanomedicine and the mechanism of PCMs controlled release in tumor therapy.

Cancer is one of the leading causes of death in the world today, and the main clinal treatments for cancer are surgery, radiotherapy, and chemotherapy (Ferlay et al., 2021). However, for surgical and radial treatments, cancer is prone to recurrence. At the same time, chemotherapy may result in certain toxic effects because of the traditional drug delivery methods of whole-body administration through vein injection (Wang and Cui, 2016; Xin et al., 2016). In order to reduce side effects and improve drug utilization efficiency, many efforts have been made in the past decades. Phase change materials (PCMs) are promising candidates for designing novel anti-cancer drug systems. PCMs is often used to encapsulate drugs into matrix material for controlling the release. Generally, the drug and PCM are mixed together first, and with the temperature change, the PCMs undergo a discontinuous phase change or morphological change to achieve drug control release. That is, the drug trapped in the solid matrix of the PCMs will be released rapidly along with the melted PCMs (Qiu et al., 2020). Due to the precise responses to the variations in temperature, PCMs can offer a simple and effective platform for antitumor smart drug delivery. As smart drug carrier in response to thermal stimulation, PCMs are fascinating for efficient tumor chemotherapy owing to the avoidance of premature drug leakage and the accurate control of site-specific drug delivery. When PCMs wrap the drug and reach the tumor site by targeting, when the tumor lesions are overheated, PCMs will undergo the solid-liquid transition to absorb heat and prevent the temperature from rising continuously. On the other hand, when the heating stops, the PCMs will release latent heat through the liquid to solid transition, avoiding a rapid drop in temperature. As a result, PCMs can maintain local temperatures close to the melting point of PCMs for relatively long periods, ensuring effective hyperthermia and/or successful release while avoiding damage to adjacent tissues. Partially benefited by the characteristics of targeting and negligible biotoxicity, controlled drug release chemotherapy using temperature-sensitive PCMs as drug gate-keeper has been proved can effectively suppress tumor growth in many studies. Recently, the nanoparticle-based delivery systems have been successfully used to overcome the accompanying difficulties of hydrophobic drugs, and the precise response of PCMs to temperature changes can endow effective drug delivery nanoplatforms for tumor therapy. In the past few decades, more and more PCMs has been designed to encapsulate drug with various nanoparticles for controllable tumor treatments. As early as 1960, the controlled release methodology had been the drug delivery research topic in the biomedical field (Hoffman, 2008), and external stimulations can control the release of drugs. Materials that respond to external stimuli, can be used as carriers for various types of payloads, such as enhancing the effectiveness and preventing the early release of payloads, which are expected to be unloaded after the stimulation when the carrier arrives at the target location. For the stimulation, it can be the temperature, PH, ultrasound, light, mechanical stress, and reactive biomolecules, et al. (Lu et al., 2016). For the PCMs used in controlling the release of the nano-encapsulated drugs, the loaded inner medicine can be released through certain conditions stimulation, and the releasing dose can be precisely controlled. Figure 1shows the types of PCMs used for intelligent antitumor administration in nanomedicine and the mechanism of PCMs controlled release in tumor therapy. What’s more, this strategy can be used for hyperthermia therapy and chemotherapy in the cancer region, improving the treatment efficiency and reducing the off-target toxicity.

Besides the control of drug encapsulation/release reviewed in this paper, PCMs have been endowed with much more advanced functions for more efficient drug delivery, such as drug protection, improving tissue distribution and cellular uptake (Li and Kataoka, 2021). PCMs can protect drugs from leaking prematurely during transport and increase cellular uptake of drugs by stimulating release after the drug enters the target cells. For example, lower critical solution temperature (LCST) polymers e.g., poly (N-isopropylacrylamide) have been used to protect nucleic acid (Li et al., 2015a). Temperature-sensitive hyperbranched polyethyleneimine derivatives increased DOX cell uptake and *in vitro* cytotoxicity at 40°C (Sideratou et al., 2018). Wrapping vitamin D in medium-chain fatty acids increases its water solubility and keeps it stable in harsh environments (Hsieh et al., 2021). Additionally, the PCMs solid matrix state is eminently suitable for protecting easily decomposed materials or drugs. Such as the small interfering Ribonucleic acid (siRNA), which has great promising in the treatment of cancer and viral infectious diseases, has poor stability in biological media and their inability to diffuse across cell membranes are huge obstacles to developing siRNA-based therapies (Zandanel et al., 2020). Yang et al. (2022a) established an intelligent nanoparticle delivery system by curing the mesoporous-mesoporous polydopamine (MPDA) and gambogic acid (GA), and siRNA with PCMs together. PCMs can effectively avoid siRNA inactivation and accidental release of GA has targeted accumulation at the tumor site. What’s more, the Bismuth (Bi), as the least toxic heavy metal, is promising in anticancer because of its unique characteristics and it is an ideal therapeutic and diagnostic agent as well. However, Bi nanoparticles are easily oxidized by oxygen in air or media, which limits their application in clinical treatment. To overcome this barrier, the Bi nanoparticles were coated with the PCM, 1-tetradecanol, which prevents the Bi oxidation in physiological environments and retains its beneficial photothermal properties. The coated PCM protects the nano-Bi from oxidation and keeps it in the Bi^0^ state for a long time (Yang L. et al., 2022). As a kind of interesting material that can accurately responded to temperature changes, multifunctional PCMs, which has abilities of ensuring that the encapsulated drugs are not decomposed, oxidized, or released early before reaching the tumor location, are novel platforms of enormous potential for drug delivery.

In light of the recent PCMs-related-nanomedicine research, this paper briefly describes the applications of PCMs in drug packaging and release. We first introduce the principal classification of PCMs’ phase transition, then we emphatically summarize the application of natural and composite PCMs. We mainly describe the technologies of PCMs’ phase transition and the controlled-release mechanisms of PCMs in tumor treatments. Eventually, this paper also discusses the potential application of PCMs in clinical tumor treatments and the future directions of PCMs-related developments.

## 2 PCMs

### 2.1 Introduce of PCMs

Generally speaking, the PCMs refer to a substance with relatively large latent heat when melting or curing at a constant temperature. The energy storage and release of nano-encapsulated PCMs have been become an important field in many applications such as electronic devices (Sahoo et al., 2016), food industry (Alehosseini and Jafari, 2019), buildings (Devaux and Farid, 2017), solar energy storage (Khan et al., 2017), aerospace (Chen et al., 2022), textiles (Prajapati and Kandasubramanian, 2019), treatment (Hyun et al., 2014) etc. In the phase transformation process, PCMs will store and release a large amount of heat within a relatively narrow range of temperature changes. Therefore, the PCMs have been widely explored for thermal management and thus can be utilized the increased fluidity during solid-liquid transitions to control the release of payloads trapped in solid matrices, resulting in smart temperature-responsive drug release systems.

### 2.2 Classification of PCMs

There are many kinds of PCMs, but the amount is limited for biomedical use as many bio-factors may influence the materials’ behaviors. PCMs can be divided into organic PCMs, inorganic PCMs, and composite PCMs according to their chemical composition. Phase change materials can absorb much heat by melting in the phase change temperature range, and most phase change materials are solid-liquid phase changes. Taking advantage of these two characteristics, they have been widely used in biomedicine in drugs delivery systems (Chen et al., 2018), biomarkers (Wang et al., 2010), medical dressings (Ma et al., 2021), and other aspects in recent years. The application of PCMs as drugs packaging materials in medical treatment can not only effectively solve the problem of drugs release but also may provide multifunctional features for tumor combined therapies. Due to space limitations, we will just focus on PCMs suitable for drug encapsulation and release. Firstly, we will discuss the natural organic PCMs’ applications in drugs encapsulation, and then the application of PCMs in the drug-controlled release of three types of composite PCMs, including the liposomes, the micelles, and the hydrogels, are introduced in detail. As shown in Table 1, the liposomes, micelles and hydrogels have different characteristics in the drug slow/controlled release (Table 1).

**TABLE 1:** Characteristics of three kinds of most common PCMs.

Types	Advantages	Disadvantages
Liposomes	Hydrophilic and oleophilic, rapid and lots of drug release in the heated area	Lack of triggers, low stability, low efficiency
Micelles	Easy prepare, small and uniform particle size, high drug load	Susceptible to decomposition and results in drug early release
Hydrogels	Good 3D mesh structure, no need for crosslinking agents, organic solvents, easy prepare	Large liquidity and cannot be the precise location

## 3 Application of natural PCMs in drug packaging

**FIGURE 2:**
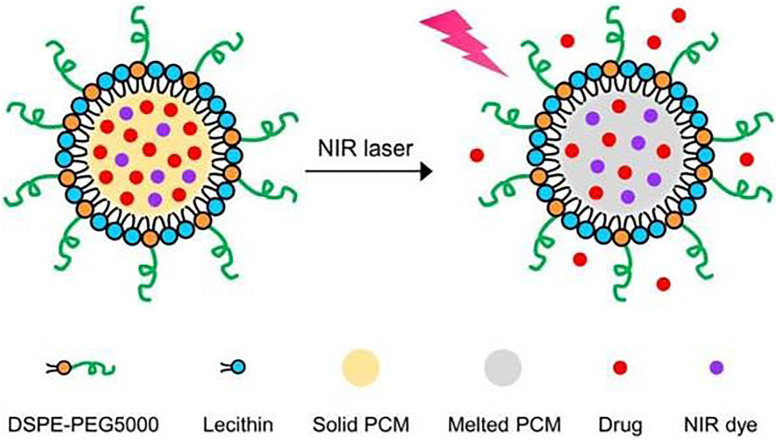
Schematic illustration showing the NIR-triggered release of drug molecules from a PCM nanoparticle made of a eutectic mixture of two fatty acids. Reproduced from Ref Chunlei et al.(2017). with permission.

PCMs has been widely used to encapsulate drugs to construct the slow/controlled release system in thermal therapy. The phase transformation nature of the PCMs is energy storage or release, which is only a reflection of the changes of intermolecular interactions (such as van der Waals forces and hydrogen bonds) (Hyun et al., 2014). Although almost all substances can undergo solid-liquid phase transformation, only a few substances are suitable for *in vivo* slow/controlled release and related biomedical applications due to the considerations of phase transition temperatures and biosafety. Moreover, blood compatibility, degradability, and other properties should also be considered. As shown in Table 2, the natural PCMs, which have been widely used in packaging drugs for slow/controlled release systems, are fatty acids and fatty alcohols. The fatty acid, which consists a long aliphatic chain, is a carboxylic acid with saturated or unsaturated fatty acids. Most natural fatty acids have a non-branched chain, and the number of carbon atoms ranges from 4 to 28, fatty alcohols have a structure similar to that of fatty acids, and they are typically straight-chain primary alcohols (Qiu et al., 2020). Because of the natural utilization, low toxicity, easy biodegradation, and low cost, natural fatty acids and fatty alcohols have been widely used in the biomedical field. Combining the PCMs encapsulated drugs with external hyperthermia stimulation is critical to implementing the synergic thermo-chemical therapy approach. Li et al. (2015b) studied the thermal response and slow/controlled drug delivery system based on novel magnetic nanoparticles using PCMs 1-tetraceanol. By wrapping 1-tetraceanol with hydrophobic paclitaxel and hydrophilic doxorubicin hydrochloride, the obtained nanosystem could precisely “turn on” or “turn off” the drug release and then, effectively, tumor cell apoptosis was observed. The system, stimulated by a high-frequency alternating magnetic field (AMF), will release drugs and then induce apoptosis by thermo-chemotherapy *in vitro* and *in vivo*. Wang et al. (2020b) used n-eicosane as PCMs and it was encapsulated in a silicon shell functionalized with acrylates. Then, by surfactant-assisted free radical polymerization, a poly (N-isopropylacrylamide-co-acrylate acid) functional layer was formatted on the silicon shell surface, and the temperature/pH dual-stimuli-response phase change microcapsule (Dual-SR-MEPCM) was synthesized. When taking Bovine serum albumin and doxorubicin hydrochloride as model drugs, Dual-SR-MEPCM showed an independent response to temperature and pH stimulus. Both drugs could be triggered by an independent low critical solution temperature and pH. Combine the natural fatty alcohols with heat generation material as a composite, after loading with an antitumor drug, the package of PCMs and drugs will transport to the tumor site. Through external stimulation, the material will generate heat and temperature increase. When the temperature reaches the phase transition temperature, the PCMs phase will change, the drug will be released, and then the synergistic therapeutic effect of thermal–chemotherapy is realized. Due to the limited categories of natural fatty acids, it is difficult to achieve the human body’s normal temperature (37°C) using single fatty acid. Thus, researchers put forward mixing two or more kinds of fatty acids with different proportions to format a temperature-appropriate composite. The melting point (MP) of the eutectic composite can be very close to the human body’s normal temperature, and the crystallization behavior of single-component fatty acids was changed accordingly, which may increase drug loading capacity. Zhu et al. (2017) (Chunlei et al., 2017) obtained a eutectic mixture with a melting point of 39°C by mixing lauric acid (MP = 44°C) and stearic acid (MP = 69°C) with a weight ratio of 4:1 (Figure 2). The eutectic mixture of two fatty acids was used as the gated material to construct the near infrared ray (NIR) response nanoparticles for drug slow/controlled release. The nanoparticles were co-encapsulated with doxorubicin (DOX) and IR780 iodide (IR780) into the eutectic mixture. The fluorescence microscope analysis showed that the DOX captured in nanoparticles can be effectively released into the cytoplasm under NIR irradiation and thereby enhancing the anticancer effects. In summary, drug delivery systems can utilize natural fatty acids and fatty alcohols, which will provide a platform for the treatment of cancer with a non-toxic, biocompatible, and easily decomposed thermosensitive drug carrier. In addition, the mixed PCMs can realize a stable and desired MP, which is not only close to the normal body temperature but also provides a new avenue for drug loading and releases with natural fatty acids and offers great promise for cancer treatment.

**TABLE 2:** MPs of different fatty acids and fatty alcohols suitable for biomedical applications.

Species	Compound	MP (°C)
Fatty acid	Dodecanoic (lauric) acid	44
	Tetradecanoic acid	54.4
	Hexadecanoic acid	63
	Octadecanoic acid	69
	11-octadecenoic acid	44
	Elaidic acid	45
Fatty alcohol	1–tetradecyl alcohol	38
	Pentadecyl alcohol	41–44
	Hexadecyl alcohol	49
	1–octadecanol	59–60

## 4 Application of composite PCMs in encapsulating drug

### 4.1 Liposomes

**FIGURE 3:**
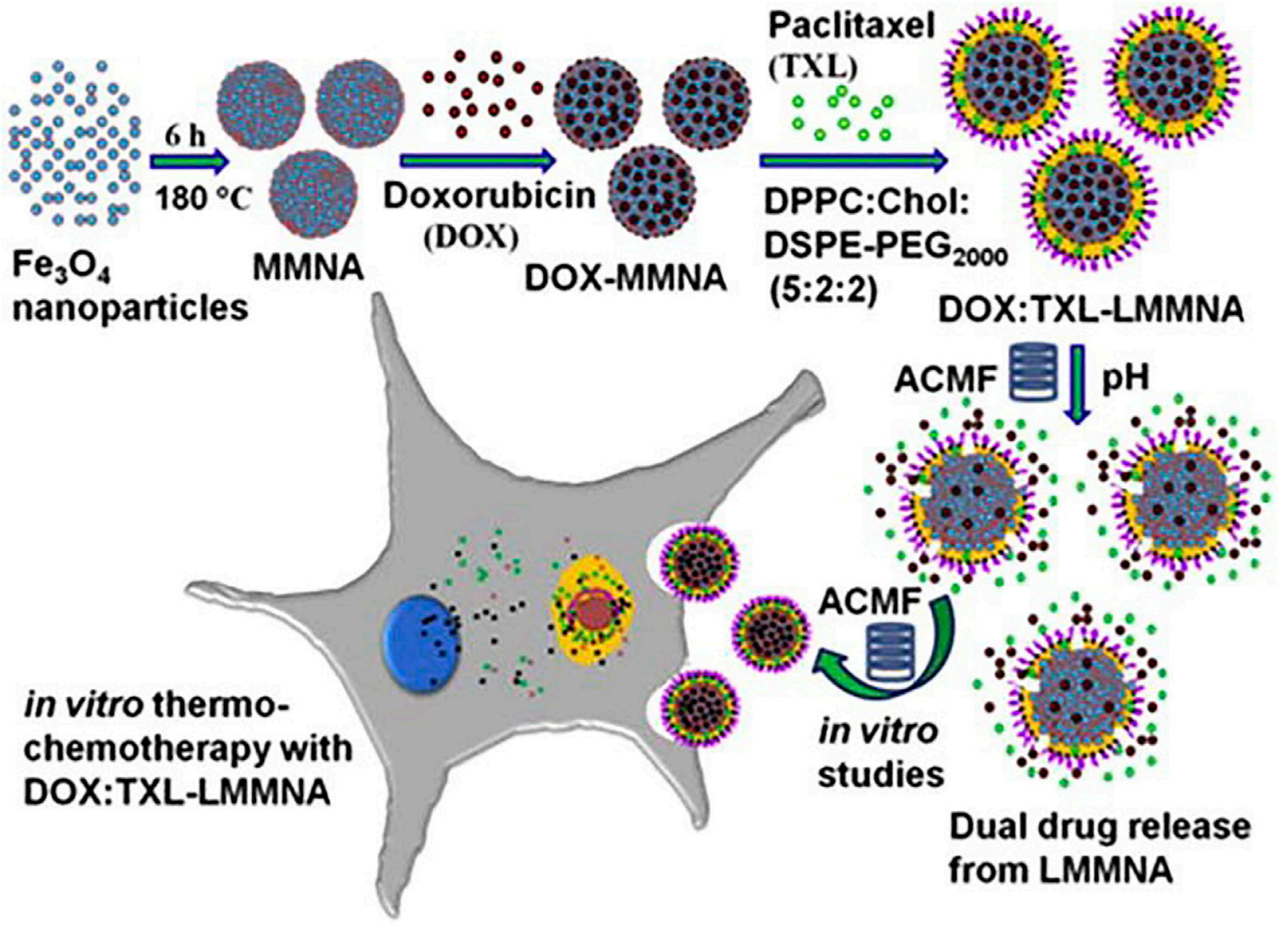
The overall concept of the present study: the pH-sensitive and thermosensitive LMMNA is a dual drug delivery system containing the drugs doxorubicin (DOX, in mesopores) and paclitaxel (TXL, in lipid layer). Drug release is triggered by an ACMF applied to the tumor cells. In this paper, the formulation and *in vitro* characterization of dual drug-loaded LMMNA with an ACMF is reported for the thermos-chemotherapy of cancer. Reproduced from Ref (Pradhan et al., 2014). with permission.

Liposomes, which are defined as phospholipid vesicles consisting of one or more concentric lipid bilayers enclosing discrete aqueous spaces, are an ideal and safe material for drug delivery (Metselaar and Storm, 2005). Liposomes are known as effective delivery systems and have been approved with lots of products and follow-on products. Liposomes are used to protect drugs from degradation in the body, control the drug release, change biological distribution, deliver the targeted drug to the focal zone and improve the solubility and bioavailability of drugs. As a drug delivery system, liposomes have been developed to be capable of loading chemotherapy drugs (Jin et al., 2016), anti-microbial and viral drugs (Guang et al., 2004), anti-parasitic drugs (Date et al., 2007), vaccines (Wang et al., 2019), therapeutic proteins (Refaat et al., 2021), anti-inflammatory drugs (Barone et al., 2020) and hormones (Dubey et al., 2006). The liposome can be divided into four categories: precursor liposome, long cycle liposome, immune liposome, and thermosensitive liposome (TSL), and this part mainly focuses on the TSL with phase change properties. TSL is a biomolecular layer where phase changes occur at a certain temperature, which is known as phase transition temperature. When the temperature is lower than the phase transformation temperature of the liposome, TSL is in the gel state, and the drug will not be released. While, when the temperature rises above the phase transformation temperature, TSL will change from the gel state to the liquid crystal state and the drug will be released. In recent years, thermosensitive liposomes have been successfully used as drug carriers for tumor treatments. Li et al. (2020) developed a targeted long-circulating thermosensitive smart-release liposome (LCTL) nanosystem, and then the reverse-phase evaporation method was used to prepare oxaliplatin (L-OHP) loaded LCTL (L-OHP/LCTL). It was found that the L-OHP was almost released from wrapped LCTL at 42 °C, while the release amount was only 10% at 37°C. The cytotoxicity of LCTL at 42 °C was much higher than that at 37°C. *In vivo* experiment results showed that the LCTL could target the tumor and remain at the tumor site for more than 24 h. Pradhan et al. (2014) developed a new pH-sensitive and thermosensitive dual drug delivery system using mesoporous magnetite nanomodules (MMNA), and lipid-coated MMNA (LMMNA) was synthesized (Figure 3). After loading with doxorubicin (DOX) and paclitaxel (TXL) in LMMNA, sustained drug release was observed for 172 h, and the final the release ratios for DOX and TXL are 72% and 68%, respectively, during the first hour of AMF presence. Additionally, the drug-loaded LMMNA was injected into the tumor site of mice, and they found that AFM would enhance the drug diffusion. The authors attribute it to local heat generated by the magnetic nanoparticles of LMMNA under AMF. In addition, due to the synergistic effect of thermo-chemotherapy, the resulting heat enhanced the effectiveness of killing tumor cells. Many studies have proved that drug candidates can be encapsulated in temperature-sensitive liposomes, which would not only be stored for a long time without external excitation but also reduce toxicity and prolong the therapeutic duration. Additionally, some thermosensitive liposomes have targeting properties, and these liposomes with specific functions can be enriched in corresponding tumor tissues and may have promising applications in further.

### 4.2 Micelles

In recent decades, temperature-sensitive micelles have attracted extensive attention from researchers. Polymer micelles are two-phase structures of components consisting of a spherical hydrophobic core and a hydrophilic shell. Micelles play a pivotal role in drug delivery (Ahmad et al., 2014). The hydrophilic segment of micelles will be exposed to solvents, and the hydrophobic segment of the micelles will be isolated from the center of amphiphilic physical assembly blocks or graft copolymers. The basic requirements for the application of micelles in drug delivery are good biocompatibility and biodegradability of micellar materials. Here, we review the applications of temperature-sensitive polymer micelles in the slow/controlled drugs. Li et al. (2014) fabricated a drug delivery system with an upper critical solution temperature of 43°C based on an amphiphilic polymer poly (AAm-co-AN)-g-PEG. This novel drug delivery system can effectively package hydrophobic anti-tumor drugs and achieve a prolonged circulation time after finishing the delivery task. What’s more, this nanocarrier can continue to regulate drug releases by controlling the temperature of the tumor, and an excellent antitumor effect was observed when microwave hyperthermia was applied. This temperature-triggered poly (AAm-co-AN) -G-PEG micelle extends the range of temperature-triggered drug delivery systems and may have the potential to be used to treat cancer. Song et al. (2017), Xia et al. (2017) developed a heat-responsive micellar drug delivery system using non-covalently connected supramolecular block polymers. The system is based on the host-guest interaction between β -cyclodextrin (β -CD) aggregating poly (n-isopropylacrylamide) star host polymer and adamantinely containing polyethylene glycol (Ad-PEG) guest polymer to form a supramolecular pseudo block copolymer called βCD-(PNIPAAm) 4/Ad-PEG. Then the micellization occurred, and drug molecules were encapsulated in the micelle core to form the final heat-responsive drug delivery system. Luo et al. (2011) prepared a dendritic monomolecular polymer micelle (H40-PDEA-PDMA) with temperature-sensitive shells through a reversible addition-fracture transfer technique. H40-PDEA-PDMA was preliminary proved with a reversible two-stage thermal particle size change during cool-heat cycles, which has great potential in various applications, like drug delivery, isolation, and gene delivery. Micelles have good biocompatibility, low toxicity, high drug loading capacity, long cycling time in blood, and appropriate size to enhance permeability and enhanced permeability and retention (EPR) effect, which is particularly beneficial for tumor therapy and thus is expected to be used as drug carriers. In a word, the thermal responsive micelles have the potential to be used in drug delivery applications to achieve superior therapeutic outcomes.

### 4.3 Hydrogels

Hydrogels are hydrophilic polymer gels that can significantly swell but not dissolve in water. In recent years, there has been increasing research on hydrogels, and hydrogels show sol-gel responses to external stimulations, like Gacanin et al. (2018), light (Dong et al., 2017), temperature (Osman et al., 2017), and movements (Zhao et al., 2020), and the temperature-sensitive hydrogel is one of the most widely used substances. For thermosensitive hydrogels, the drugs are loaded onto hydrogels, and temperature changes can control the hydrogels’ state. Through chemical crosslinking and physical interaction, a variety of thermosensitive hydrogels for drug delivery have been developed (Yin et al., 2021). Wu et al. (2020) synthesized an mPEG-PLGA-BOX block copolymer hydrogel, which was found had good biocompatibility and low toxicity. Interestingly, it can be directly injected into the tumor site in a liquid state at a low temperature. Then use a 0.4 W/cm^2^ ultrasound scan to heat the hydrogel into a solid state, which would keep the material in the desired position and could not flow to other parts of the body, and release the drug into the same region for the chemical therapy. Madry et al. (2020) prepared an injectable hydrogel based on PEO-PPO-PEO copolymer, which can control the release of therapeutic recombinant adeno-associated virus (rAAV) vector overexpressed in full-layer cartilage defects. PEO-PPO-PEO copolymers are non-ionic triblock copolymers based on hydrophilic poly (ethylene oxide) (PEO) and hydrophobic poly (propylene oxide) (PPO). These copolymers have self-assembly and temperature sensitivity, such as the individual block copolymers (monomers) can self-assemble into micelles at concentrations above critical micelle concentration (CMC). Similarly, by increasing the temperature and concentration, the micelles will form a highly viscous 3D network (gel) and form a sol-gel transition state at about 37°C, which can be injected as a liquid at low temperature and becomes a semisolid gel at high temperature, and enable a controlled release of drugs at the implantation site. Thermosensitive hydrogels based on PEO-PPO-PEO polymers, which can control the release of therapeutic rAAV vectors, were proved could significantly improve the repair of full-thickness cartilage defects in animal models. Wu et al. (2018) fabricated an injectable and thermal-responsive supramolecular poly (N-acrylamido-co-acrylamide) (PNAm) hydrogels to load polydopamine (PDA) coated gold nanoparticles (AuNP) and doxorubicin (DOX). After heating with the near-infrared laser irradiation, the hydrogel could be injected into the cavity of postoperative breast cancer by inducing gel-sol transformation. The built-in photothermal effect promoted the drug release from the PNAm-PDAAu-DOX hydrogel due to the dissociation of the physical cross-linking. Moreover, the photothermal effect of PNAm-PDAAu hydrogel remained stable after five cycles of light on/off switches, and the drug release and local heating could be achieved by irradiating the nanocomposite hydrogel with near-infrared light. In recent years, thermosensitive hydrogels proved a great success in cancer treatments (Zhang and Song, 2016; Zheng et al., 2019). By injecting the drug carrier hydrogel injection by to the tumor site, this approach could provide high local drug concentration, slow-release characteristics, minimally invasive, and low systemic toxicity. Thermosensitive phase change hydrogels provide a new drug carrier and repair function platform.

## 5 Method of controlled release of drugs

PCMs can control the release of the encapsulated drugs by direct heating, photothermal, magnetothermal, microwave heating and other methods. We can combine the materials, which have excellent effects of photothermal, magnetothermal, and microwave, with PCMs, the PCMs phase will change when applying the corresponding stimulus and drug release consequently. The synergistic treatments of thermos-chemical therapy can achieve a significantly enhanced tumor therapeutic effect. This section will focus on the different external stimuli and the mechanism of PCMs-based slow/controlled system-controlled release in tumor therapy.

### 5.1 Direct heating method

PCMs undergo a reversible phase transition in response to temperature changes. To this end, direct heating is the most common method to change temperature and then trigger the solid-liquid transition. Direct heating is also a convenient way to evaluate the controlled release effects of PCMs in the laboratory. As for *in vivo* applications, direct heating can be applied to the local skin by various methods and devices, but it is unsuitable for treating deep tissue diseases. On the other hand, the increased temperature in some disease sites can be used as a pathological stimulus to trigger drug release. However, it is difficult to accurately control the release curve based on the internal temperature changes. In most cases, externally induced rapid local temperature increases at the target site are more useful in triggering and controlling drug release.

### 5.2 Photothermal- chemical therapy

Photothermal therapy (PTT) is based on photothermal conversion materials, which have relatively high photothermal conversion efficiency, and the light energy can be converted into heat energy with illumination (Ray et al., 2012). Due to the advantages of noninvasive and remote temporal/spatial controllability, near-infrared NIR light has been widely used in realizing drug-controlled release. In addition, NIR light can penetrate relatively deep into soft tissues because of the reduced scattering and absorption compared to other light wavelengths. Currently, photothermal combined chemotherapy is the most widely used and studied method, and many novel nanomaterials have been synthesized for combination therapy. The PCMs temporarily encapsulated the drug, and irradiation converted the light energy into heat energy. The PCMs underwent phase change, the drug-loaded material was released in the irradiation regions, and the combined therapy was realized simultaneously. Zhou et al. (2013) developed a photothermal agent with good biocompatibility and high efficiency. He modified the surface of polyaniline nanoparticles (PANPs) with polyoxyethylene chain by hydrothermal method, and then a polyoxyethylene chain coating agent (F-PANPs) was obtained. It was found that the molar extinction coefficient of F-PANPs was 8.95 × 108 m^−1^cm^−1^, and the photothermal conversion efficiency of NIR could reach 48.5%. After irradiation with 808 nm NIR, the *in vivo* results showed that photothermal could totally ablate the tumor. Yan et al. (2016), Fei et al. (2016) prepared an intelligent DOX/IR-780 loaded temperature-sensitive liposome (DITSL) that could achieve NIR-laser-controlled drug release for photothermal-chemo combined therapy. In this system, the fat-soluble IR-780 was packaged in the lipid temperature-sensitive bilayer, while the soluble chemotherapeutic doxorubicin (DOX) was encapsulated in the hydrophilic core. The NIR-laser irradiation can induce local high temperatures (above 50°C), making the central tumor necrosis region. By destroying the temperature-sensitive liposome shell, DITSL releases DOX in the peripheral or transition zone of the tumor from 41 to 45°C. The chemotherapy combined with photothermal therapy dramatically improved the antitumor efficacy. On the other hand, the penetration of NIR light is limited to a few inches because tissue inevitably scatters and absorbs, which makes it difficult to heat the deep areas. In addition, the NIR-induced heating often requires the presence of photothermal agents, which may increase the complexity of a PCMs-based hybrid system.

### 5.3 Magnetothermal-chemical therapy

Magnetothermal responded chemical therapy is to combine the magnetic nanomaterials with PCMs and antitumor drug together, and the magnetic nanomaterials will generate heating under an AMF. Through the magnetothermal effect, the temperature change will lead to the phase transition of PCMs. The packaged drug will be released for the combined thermal therapy and chemical therapy. What’s more, the magnetic hyperthermia technique has great promise in treating deep tumors because heat is generated only in areas where magnetic nanoparticles are present, and the magnetic field can penetrate the entire human body. Faras et al. (2020) synthesized a novel magnetic liposome with a diameter of about 198 nm by the thin layer hydration method. Due to the superparamagnetic behavior, the liposome has magnetically induced heat capacity, and the phase transition temperature of the lipid bilayer is biocompatible. By applying AMF, magnetic nanomagnetic particles can heat the liposome to a certain temperature, and then the carrier will release the drug immediately. Wang et al. (2020) prepared magnetic nanoparticle (manganese zinc ferrite) as core and mesoporous silica as shell nanospheres (MMSN-RBITC), by labeling with rhodamine isothiocyanate B (RBITC), it can be fully dispersed into self-healing hydrogel system. They found that MMSN-RBITC could effectively enhance the self-healing hydrogel system. The heat generated by MMSN-RBITC under the AMF could cause the hyperthermia effect of Magnetothermal therapy and might also promote the sensitivity of chemotherapy. After MMSN-RBITCs were phagocytized by cells, hydroxyl radicals could be generated by Fenton-like catalysis, which further promoted cell apoptosis through chemo-dynamic therapy. This strategy of loading DOX in MMSN-RBITC and then mixing it with self-healing hydrogel could realize tumor diagnosis and effectively triple (magnetothermal, chemical, and chemo-dynamic) therapy. Similar to NIR, heat generation in alternating magnetic fields is highly dependent on magnetic nanomaterials, which will increase the complexity of PCMs-based release systems.

### 5.4 Microwave and chemical thermotherapy

Microwave (MW) induced hyperthermia therapy is a treatment method emerging in recent years. MW sensitizer injected into the tumor site is irradiated by microwave to generate heat and induce cell apoptosis. The MW-based thermal mechanism is that with the MW radiation, ions constantly vibrate and rapidly clash in a limited space to generate heat (Qi et al., 2019; Li T. et al., 2020). In recent years, MW-induced hyperthermia has become a powerful thermal ablation technology with good biocompatibility and technology for controlling local tumors. The combination of MW-induced hyperthermia and chemotherapy have a better therapeutic effect than the single MW or chemotherapy. Tang et al. (2017), Shunsong et al. (2017) designed a biocompatible and degradable methoxy polymer (ethylene glycol) polymer (lactic co-glycolic acid) microcapsule with a hierarchical structure for MW-induced tumor therapy. The chemical anti-cancer drug DOX, MW sensitizer, CT imaging contrast agent molybdenum disulfide nanosheets, and MRI contrast agent Fe_3_O_4_ nanoparticles were combined into the microcapsules. After the tumor site injection, focused MW has been applied to heat the WM sensitizer, and the increased temperature is damaged, which will lead the chemotherapy drug release. Long et al. (2016) designed a multifunctional nanoplatforms to enhance tumor inhibition by loading IL, DOX, and PCMs into hollow zirconia (ZrO_2_) nanoparticles. The effects of microwave and chemical thermotherapy were explored on H22 tumor-bearing mice through tail vein injection of prepared IL-DOX-PCMs@ZrO_2_ nanoplatforms. After mild microwave irradiation (0.9 W, 450 MHz), the thermal effect of MW could increase the temperature of the tumor site (58°C). Meanwhile, when the temperature increases to a certain extent, the phase transformation of PCMs would occur, and the drug will be released and the therapeutic effect of thermalization can be achieved. Although the microwave combination of the chemical thermotherapy is currently in the research stage, they are promising for applications in many biomedical fields (Tang et al., 2016).

## 6 Summary

This review summarized different PCMs, including the natural fatty acids, fatty alcohols, liposomes, micelles, and water gel and their applications in drugs encapsulation, delivery, and slow/controlled release. We also introduce the triggers for PCMs in treating tumor drug-controlled release methods, like direct heating, photothermal, magnetothermal, and microwave heating. The phase change temperature of PCMs is close to the body temperature and can be used as drug carriers for disease treatments. The higher phase change temperature can not only achieve a good therapeutic effect but may also damage the normal tissues, while the lower phase change temperature can only be used for single drug treatment and cannot achieve the hyperthermia therapeutic effect. As drug encapsulating material, PCMs must have properties such as good biocompatibility, low toxicity, and easy decomposition. In recent years, compound PCMs becomes the research hotspot, including liposomes, micelles, and hydrogels. The compound PCMs have better performance and structure than natural PCMs, which can meet the corresponding requirements by changing the synthesis process. PCMs is becoming more and more important in medical applications, and the method of combining hyperthermia and drug therapy is more intelligent and less toxic than the traditional single treatment. Additionally, the hyperthermia trigger is not only limited to single photo, but also more and more other methods, such as magnetic hyperthermia and microwave hyperthermia. Combining PCMs with multifunctional nanomaterials allows for more effective cancer treatments through a variety of therapeutic approaches. Despite the success of PCMs-based drug delivery systems, many challenges still require additional attention in the future. For example, the toxicity and safety of nanomaterials for *in vivo* applications remain uncertain. What’s more, most nano-drug delivery systems are not suitable for commercial production due to their complex preparation process, high development cost, and low encapsulation rate. It is believed that in the near future, the application of PCMs is not limited to research, but will be more practical and can be widely applied in human diseases treatments.
